# Phase I trial of oral S-1 combined with gemcitabine in metastatic pancreatic cancer

**DOI:** 10.1038/sj.bjc.6602644

**Published:** 2005-06-07

**Authors:** K Nakamura, T Yamaguchi, T Ishihara, A Kobayashi, H Tadenuma, K Sudo, H Kato, H Saisho

**Affiliations:** 1 Department of Medicine and Clinical Oncology, Graduate School of Medicine, Chiba University, Chiba, Japan; 2 Research Center Hospital for Charged Particle Therapy, National Institute of Radiological Sciences, Chiba, Japan

**Keywords:** S-1, gemcitabine, metastatic pancreatic cancer, phase I study

## Abstract

The objective of this study was to determine the maximum tolerated dose (MTD) and dose-limiting toxicities (DLTs) of S-1, an oral fluorouracil derivative, combined with gemcitabine, the current standard treatment for advanced pancreatic cancer (APC). The subjects were histopathologically proven APC patients with distant metastasis. S-1 was administered orally twice daily each day for 14 days and gemcitabine on days 8 and 15 of each cycle, and this was repeated every 21 days. Doses of each drug were planned as follows: level 1: 800/60, level 2a: 800/80, level 2b: 1000/60, level 3: 1000/80 (gemcitabine (mg m^−2^)/S-1 (mg m^−2^ day^−1^)). In all, 21 patients with APC were enrolled. The main grade 3–4 toxicities observed during first cycle were neutropenia (33%), anaemia (10%), thrombocytopenia (14%) and anorexia (10%). There were no DLT observed in level 1. Three of six patients in level 2a had DLT and this level was considered the MTD. In all, 12 patients in level 2b had no DLT and this level was selected as the recommended dose. Applicable responses were one complete response and nine partial responses (48%). As toxicities were well tolerated and antitumour activities seem to be promising, this combination can be recommended for further phase II studies with APC.

The incidence and mortality of pancreatic cancer has increased so rapidly over the past 20 years in Japan that it is now the fifth leading cause of cancer mortality in the country (Matsuno *et al*, 2004). The 5-year survival rate is still poor, at less than 10%, commonly considered to be linked to the high incidence of distant metastasis even at initial diagnosis, as well as the tumour's resistance to anticancer agents. Innovation in systemic chemotherapy is thus urgently needed to improve the survival of patients with pancreatic cancer (Glimelius *et al*, 1996; Evans *et al*, 1997).

Since 1997, gemcitabine has been the most widely used chemotherapeutic agent in advanced pancreatic cancer (APC) and was reported to have significantly better symptom control in APC compared with 5-FU in a randomised phase III clinical study (Burris *et al*, 1997). Even with gemcitabine, however, mono-therapy has obvious limitations in APC and various combinations with other agents have been investigated. The combination of gemcitabine and 5-FU is shown to have a marked synergistic cytotoxic effect against pancreatic cancer cells in *in vitro* assay (Bruckner *et al*, 1998). Phase I and II studies of combined therapy of gemcitabine with 5-FU demonstrated superior results (Berlin *et al*, 1998, 2000; Cascinu *et al*, 1999; Hidalgo *et al*, 1999; Matano *et al*, 2000). However, adding weekly intravenous bolus 5-FU to weekly gemcitabine did not confer a significant survival benefit in a randomised trial (Berlin *et al*, 2002). There are no randomised data on the combination of infusional 5-FU with gemcitabine in APC.

S-1 is a new oral fluorinated pyrimidine developed by Taiho Pharmaceutical Co. Ltd (Tokyo, Japan). The agent contains tegafur (FT), 5-chloro-2,4-dihydroxypyridine (CDHP) and potassium oxonate (Oxo) in a molar ratio of FT : CDHP : Oxo=1 : 0.4 : 1, based on a biochemical modulation of 5-FU (Shirasaka *et al*, 1996a, 1996b). Tegafur, a prodrug of 5-FU, is gradually converted to 5-FU and is rapidly catabolised by dihydropyridine dehydrogenase (DPD) in the liver. 5-Chloro-2,4-dihydroxypyridine is a competitive inhibitor of 5-FU catabolism, being about 180 times more potent than uracil in inhibiting DPD (Tatsumi *et al*, 1987). When tegafur is combined with CDHP, the resulting high 5-FU levels are maintained in both plasma and tumour. In addition, it has been suggested that CDHP has the potential to enhance the antitumour activity of 5-FU against subcutaneous tumour in nude mice, using human pancreas carcinoma cells with a high tumoral DPD activity (Takechi *et al*, 2002). Oxo inhibits the enzyme orotate phosphoribosyltransferase, the major enzyme responsible for 5-FU activation in colon cancer (Peters *et al*, 1991). Oxo preferentially localises in the gut rather than in the tumour and has a potential biochemical effect on the enzyme orotate phosphoribosyltransferase, thereby selectively inhibiting the formation of 5-FU nucleotides in the gut and theoretically reducing gastrointestinal side effects (Takechi *et al*, 1997). In phase II studies for advanced gastric cancer conducted in Japan, S-1 showed high response rates of 44–49% (Sakata *et al*, 1998; Koizumi *et al*, 2000), and the usefulness of S-1 was also reported in head and neck (Inuyama *et al*, 2001), breast (Saeki *et al*, 2004) and colorectal cancer patients (Ohtsu *et al*, 2000). In studies outside Japan, the phase II studies of S-1 against gastric (Chollet *et al*, 2003) and colorectal cancer (Van den Brande *et al*, 2003) in Europe by the EORTC-Early Clinical Study Group revealed moderate activity. The antitumour activity of S-1 in patients with pancreatic cancer has not yet been investigated outside Japan, but preliminary favourable results of S-1 have been reported in Japanese early phase II study of patients with APC (Okada *et al*, 2002).

The administration of oral S-1 is more convenient and simulates the effect of continuous infusion of 5-FU. We anticipated that combination chemotherapy of gemcitabine and S-1 would be effective through the additive and synergistic activity of gemcitabine and 5-FU derived from S-1. As yet, the combination regimen of gemcitabine and S-1 for patients with APC has not been investigated. Therefore, the author performed a phase I study to evaluate the safety of treatment combined gemcitabine with S-1 and to determine the maximum tolerated dose (MTD) of each drug for patients with APC.

## PATIENTS AND METHODS

### Patient selection

Patients with histopathologically proven APC with distant metastasis were eligible for the study. Other eligibility criteria included: 20–74 years of age, Eastern Cooperative Oncology Group (ECOG) performance status of 2 or less (ambulatory and capable of self-care), estimated life expectancy of more than 2 months, adequate renal function (normal serum creatinine and blood urea nitrogen levels), liver function (total bilirubin level ⩽2.5 times upper normal limit (UNL) or ⩽3 times UNL after biliary drainage if the patient had obstructive jaundice and serum transaminases (GOT, GPT) levels ⩽2.5 times UNL or ⩽3 times UNL), bone marrow reserve (white blood cell count between 4000 and 12 000 mm^−3^, neutrophil count ⩾2000 mm^−3^, platelet count ⩾100 000 mm^−3^ and haemoglobin level ⩾9.5 g dl^−1^) and pulmonary function (PaO_2_⩾70 mmHg). If the patients had a previous history of cancer treatment, that treatment (tumour resection, chemotherapy, immunotherapy, or radiotherapy) had to have been discontinued for at least 4 weeks before entry into the study. All subjects provided written informed consent.

The exclusion criteria were as follows: pulmonary fibrosis or interstitial pneumonia, marked pleural or pericardial effusion or marked peripheral oedema, severe heart disease, difficult to control diabetes mellitus, active infection, pregnant or lactating females, women of childbearing age unless using effective contraception, severe drug hypersensitivity, metastases to the central nervous system, severe neurological impairment or mental disorder, active concomitant malignancy and other serious medical conditions.

This study was approved by the institutional review board of Chiba University Graduate School of Medicine.

### Study design

This was an open-label, single-centre, nonrandomised, dose-escalating phase I study. All laboratory tests required to assess eligibility had to be completed within 7 days prior to the start of treatment. S-1 was administered orally twice daily after a meal for 14 consecutive days (from the evening of day 1 to the morning of day 15), followed by a 1-week break. Each capsule of S-1 contained 20 or 25 mg of tegafur. Individual doses were rounded down to the nearest pill size less than the calculated dose, given the available formulation. Gemcitabine was administered as a 30-min intravenous infusion on days 8 and 15 of each cycle. The cycle was repeated every 21 days. This schedule was based on an *in vitro* study which showed maximum synergy when fluoropyrimidine precedes exposure to gemcitabine (Rauchwerger *et al*, 2000). The dose of each drug in this study was planned as follows: level 1 was S-1 60 mg m^−2^ day^−1^ and gemcitabine 800 mg m^−2^, level 2a was S-1 80 mg m^−2^ day^−1^ and gemcitabine 800 mg m^−2^, level 2b was S-1 60 mg m^−2^ day^−1^ and gemcitabine 1000 mg m^−2^, level 3 was S-1 80 mg m^−2^ day^−1^ and gemcitabine 1000 mg m^−2^. However, only when neither level 2a nor level 2b reached the MTD would patients be assigned to dose level 3.

### Definition of dose-limiting toxicities (DLTs) and MTD

Dose-limiting toxicities (DLTs) were determined during the first treatment cycle. Dose-limiting toxicity was defined, using the National Cancer Institute (NCI) Common Toxicity Criteria (CTC) scale (version 2.0), as one or more of the following effects attributable to study drug: (a) grade 3 or 4 neutropenia complicated by fever; (b) grade 4 neutropenia lasting longer than 4 days; (c) grade 4 thrombocytopenia; (d) any other grade 3–4 nonhaematologic toxicity except anorexia, nausea and vomiting in the absence of appropriate antiemetics and (e) delay of recovery from treatment-related toxicity for more than 2 weeks. At least three patients were enrolled at each dose level. If DLT was observed after the first cycle in one or two patients, three additional patients were placed on that dose level. If only one or two of six patients experienced DLT, dose escalation would continue. There was no dose escalation in individual patients. The MTD of the combination was defined as the dose level that produced DLT in ⩾3 of six patients or in all of the initial three patients. The recommended dose (RD) was defined as the dose level that is one level under MTD considering the toxicity and tolerability in outpatient setting.

### Pretreatment and follow-up studies

Before entry into the study, all patients gave a full history and underwent a physical examination. A complete blood count (CBC) with differential, electrolyte levels, and creatinine levels were measured. Routine chemistry tests, urinalyses and 24-h urine collections were performed to detect proteinuria. Electrocardiograms, chest X-rays and computed tomographic scans of the chest and abdomen were performed at baseline in all patients. Additional imaging investigations were performed if clinically indicated or for disease measurement. A complete blood count with differential, serum chemistry, creatinine level, and electrolyte level were measured weekly. Computed tomographic scanning and imaging of the measurable disease to assess tumour response were performed every two cycles. At the completion of the study, all clinical, laboratory, radiologic imaging and other evaluations were repeated. After completion of the study, patients underwent follow-up examinations every 2 months until death. Additional treatment after disease progression was left to the discretion of the treating physician.

### Assessment of efficacy

All patients were included in efficacy measurements on an intent-to-treat basis. Tumour responses were evaluated according to the World Health Organization's criteria (World Health Organization, 1979). A complete response (CR) was defined as the disappearance of all evidence of cancer for 4 weeks or longer. A partial response (PR) was defined as a 50% or more reduction in the sum of the product of the longest perpendicular dimensions of all lesions for 4 weeks or longer without any evidence of new lesions or the progression of any lesions. Stable disease (SD) was defined as less than a 50% reduction or less than a 25% increase in the sum of the product of the longest perpendicular dimensions of all lesions without any evidence of new lesions. Progressive disease (PD) was defined as a greater than 25% increase in one or more lesions or the appearance of any new lesion. To assess objective response, patients were evaluated every 6 weeks (two cycles) by three independent radiologists.

Serum CA19-9 levels were measured every 4 weeks during the chemotherapy using a commercially available chemiluminescent enzyme immunoassay based on the two-step sandwich method (CL-EIA). A value of 37 U ml^−1^ was defined as the upper limit of the normal.

Overall survival was estimated from the date of first treatment to death or last follow-up visit, calculated using the Kaplan–Meier method, and confidence intervals (CI) were based on Greenwood's formula.

## RESULTS

All 21 patients with APC registered between January 2003 and March 2004 had primary sites. Out of 21, 18 patients had liver metastasis except one who had lung metastasis, and two who presented with peritoneal carcinomatosis only (Table 1). Although the eligibility criteria included patients who had a previous history of cancer treatment (tumour resection, chemotherapy, immunotherapy, or radiotherapy) before entry into the study, in actuality no patients had previously received such treatment.

The numbers of patients at each level are shown in Table 2. Three patients were assigned to dose level 1 without DLT. At dose level 2a, DLT was observed in two of the first three patients; thus three additional patients were assigned to this level. Dose-limiting toxicity was observed in three of six patients, and level 2a reached MTD. Thus, three patients were assigned to level 2b and no DLT was observed in the first three patients. However, nine additional patients were assigned to this level to explore the responses to and continuity of the treatment.

### Toxicity and treatment cycles

The most common toxicities observed during the first cycle of chemotherapy are listed in Tables 3 and 4. Of three patients in level 1, one had thrombocytopenia of grade 3, but no DLT leading to MTD was observed in any patient. Of six patients in level 2a, grade 3–4 neutropenia occurred in four patients, grade 3 anaemia in one patient and grade 3 thrombocytopenia in two patients. In terms of nonhaematological toxicities, grade 4 anorexia, grade 3 nausea and grade 3 rash occurred in one patient, each. Three of six patients at level 2a showed DLT; one patient developed sepsis with grade 4 leukopenia and neutropenia, a second patient developed a grade 3 rash and a third patient developed grade 2 leukopenia, not recovering within the planned period. Thus, DLT was observed in three of six patients, and level 2a reached MTD. Of 12 patients at level 2b, grade 3 to 4 neutropenia occurred in three patients and grade 3 anaemia in one patient, while grade 3 anorexia occurred in one patient, and DLT leading to MTD was not observed. Based on these results, level 2b was selected as the RD for the phase II study we are to conduct.

The median and range of the treatment cycles and the number of patients who received a dose reduction were shown in Table 5. The median number of cycles delivered at dose level 2b, which was selected as the RD, was four, and only six of 61 cycles at this dose level needed to reduce their dose of gemcitabine.

### Efficacy

Although assessment of tumour response was not a primary objective of this study, patients were evaluated for tumour response every two cycles (6 weeks) of the treatment. All 21 patients were assessed for response during this treatment. Responses in the 21 assessable patients were: one CR (dose level 2a), nine PRs (one at dose level 1, three at dose level 2a and five at dose level 2b), six stable disease (two at dose level 1, one at dose level 2a, and three at dose level 2b) and progression in only five patients (one at dose level 2a and four at dose level 2b). As a result, 10 of the 21 patients (48%) showed complete or PRs (Table 6). The value of CA 19-9 before treatment was elevated (>37 U l^−1^) in 15 of 21 patients. Of those 15 patients, CA 19-9 decreased 50% or more compared with the level prior to treatment in seven (47%) and showed a normal value in three (20%). In contrast, an increase of CA 19-9 was observed in only four patients (27%). At present, seven patients are still alive. After a median follow-up of 8.9 months (range, 2.2–16.1 months), the median survival time was 9.3 months (95% CI, 6.3–12.3%) and the 1-year survival rate was 35% (95% CI, 12–58%).

## DISCUSSION

The primary end point of this trial was to define a chemotherapy regimen with an acceptable toxicity profile that could potentially improve the therapeutic efficacy of gemcitabine in patients with pancreatic cancer. S-1 has been selected as a candidate to be investigated in combination with gemcitabine in patients with pancreatic cancer because of its consistent activity as a single agent in this disease and because of the lack of cross-resistance between gemcitabine and 5-FU derived from S-1, as suggested by the observed activity of gemcitabine in patients refractory to 5-FU (Rothenberg *et al*, 1996). Also, gemcitabine combined with infusional 5-FU has been noted to possess synergy in *in vitro* cytotoxicity in a variety of malignant cell lines, including pancreatic cancer (Bruckner *et al*, 1998). Therefore, we expected additive and synergistic efficacy by combining gemcitabine with S-1, hoping that it would mimic the continuous infusion of 5-FU and also have DPD inhibition, leading to enhancement of antitumour activity (Takechi *et al*, 2002).

When considering this study regimen, the authors considered the possibility that this combination of gemcitabine with S-1 might produce more severe toxicities than those generated by gemcitabine alone. Thus, we tried to lessen the frequency of gemcitabine in this regimen, administering it twice every 3 weeks. S-1 has already undergone phase I and II testing in several solid tumours in Japan and western countries. The DLT was myelosuppression in a Japanese phase I study (Taguchi *et al*, 1997), and diarrhoea in a European and a North-American phase I study (van Groeningen *et al*, 2000; Hoff *et al*, 2003). In Japan, the standard single-agent dose is 80 mg m^−2^ day^−1^ for 28 consecutive days, every 5–6 weeks, although the RD of S-1 was 70–80 mg m^−2^ for 28 consecutive days, every 5 weeks in Europe, and 60 mg m^−2^ for 28 consecutive days, every 5 weeks in the US, divided into twice-daily doses. Consequently, we conducted this study in an attempt to maintain the same dose intensity as that used in the standard S-1 administration, but in combination with gemcitabine. Both of the phase II trials in Japan revealed that low grades of gastrointestinal toxicities, including nausea, vomiting and anorexia, and of myelotoxocities such as neutropenia, occurred frequently during the third week of S-1 administration. Therefore, we adopted the regimen of S-1 administration for 14 consecutive days repeated every 3 weeks to avoid severe toxicity. The dose intensity of S-1 in this regimen amounts to almost the same level as that in Japanese standard regimen: S-1 for 28 consecutive days, every 5–6 weeks. Also, given that an *in vitro* study of pancreatic cancer cells has also demonstrated maximum synergy for gemcitabine when exposure to a thymidylate synthase inhibitor such as 5-FU precedes exposure to gemcitabine (Rauchwerger *et al*, 2000), we adopted the regimen of gemcitabine administration on days 8 and 15 after S-1 administration of each cycle.

Myelosuppression, especially neutropenia, frequently seen in the combination of continuous infusion 5-FU and gemcitabine, was predicted as the main toxicity of this study. In this study, the incidence of grade 3 or 4 neutropenia during the first cycle was higher than that of other toxicities, with four of six patients at dose level 2a and three of 12 patients at dose level 2b having grade 3 or 4 neutropenia. On the other hand, the incidence of gastrointestinal toxicity during the first cycle and all cycles was low. Only one patient at dose level 2a had grade 4 anorexia and grade 3 nausea, one patient at dose level 2b had grade 3 anorexia.

A median number of 10 cycles were administered at dose level 1, seven cycles at dose level 2a and four cycles at dose level 2b. However, there was no significant difference among the median number of administered cycles at every dose level. During all treatment cycles in this study, the incidence of grade 3 or 4 neutropenia at dose level 2b was 10%, at dose level 1 it was 19%, and at dose level 2a it was 33%. Consequently, only six of 61 cycles at dose level 2b needed a dose reduction of gemcitabine compared to 31 of 66 cycles at dose level 2a, which required that.

The first course of chemotherapy was conducted by hospitalisation for all patients, but the second or subsequent courses could be performed at an outpatient clinic for 19 of 21 patients. The other two patients showed early progression of the disease. Moreover, oral administration of S-1, which eliminates the cost and inconveniences of infusion pumps and catheters with their potential risks of infection and thrombosis, also contributes to fewer hospital visits during this outpatient treatment. Anticancer treatment for APC would be preferable on an outpatient rather than an inpatient basis, given the short life expectancy and quality of life considerations. In treatment for patients with APC, it is important to not only improve the prognosis of APC but also create a feasible regimen of chemotherapy that does not require hospitalization. These results indicated that the combination at the RDs selected in this study is quite feasible in the outpatient treatment setting.

In conclusion, this combination chemotherapy with gemcitabine and S-1 was well tolerated. Although this trial was only a phase I study to determine the RD and feasibility of such combination, an encouragingly high response rate has been observed. This result is very promising, but the survival benefit in comparison with gemcitabine monotherapy needs to be confirmed in future studies.

## Figures and Tables

**Table 1:** Patient characteristics

Patients enrolled	21
Men	10
Women	11
*Age, years*	
Median	61
Range	48–73
	
*ECOG status*	
0	8
1	11
2	2
	
*Sites of metastatic disease*	
Liver	18
Lung	3
Peritoneum	2

**Table 2:** Dose levels

**Dose level**	**S-1 (mg m^−2^ day^−1^: 2 weeks)**	**Gemcitabine (mg m^−2^:** **on days 8, 15)**	**No. of patients**
1	60	800	3
2a	80	800	6
2b	60	1000	12

**Table 3:** Haematological toxicity during first cycle (in all cycles)

		**No. of patients (cycles) with grade of toxicity**						
		**Neutropenia**		**Anaemia**		**Thrombocytopenia**		
**Dose level**	**Total no. of patients (cycles)**	**1–2**	**3–4**	**1–2**	**3–4**	**1–2**	**3–4**	**DLT**
1	3 (27)	2 (13)	0 (5)	3 (5)	0 (1)	2 (6)	1 (5)	
2a	6 (66)	2 (34)	4 (22)	5 (15)	1 (5)	4 (13)	2 (13)	2
2b	12 (61)	7 (25)	3 (6)	5 (8)	1 (2)	11 (13)	0 (0)	

**Table 4:** Nonhaematological toxicity during first cycle (in all cycles)

		**No. of patients (cycles) with grade of toxicity**						
		**Anorexia**		**Nausea and vomiting**		**Rash**		
**Dose level**	**Total no. of patients (cycles)**	**1–2**	**3–4**	**1–2**	**3–4**	**1–2**	**3–4**	**DLT**
1	3 (27)	1 (2)	0 (0)	1 (1)	0 (0)	3 (6)	0 (0)	
2a	6 (66)	1 (4)	1 (2)	1 (5)	1 (2)	3 (7)	1 (1)	1
2b	12 (61)	2 (5)	1 (1)	5 (8)	0 (0)	11 (12)	0 (0)	

**Table 5:** Duration of administration and dose intensity

			**No. of cycles**		**Cycles with dose reduction in gemcitabine**	
**Dose level**	**S-1/gemcitabine (mg m^−2^)**	**No. of patients**	**Total**	**Median (range)**	**No.**	**%**
1	60/800	3	27	10 (3–14)	5	19
2a	80/800	6	66	7 (2–20)	31	47
2b	60/1000	12	61	4 (2–10)	6	10

**Table 6:** Objective tumour response

		**Response**				
**Dose level**	**No. of patients**	**CR**	**PR**	**SD**	**PD**	**Response rate (%)**
Level 1	3	0	1	2	0	33
Level 2a	6	1	3	1	1	67
Level 2b	12	0	5	3	4	42
						
Total	21	1	*9*	*6*	*5*	48
